# Supplementary material for: Liver transplant waitlist removal, transplantation rates and post-transplant survival in Hispanics
Source: PLoS One. 2020 Dec 31;15(12):e0244744. doi: 10.1371/journal.pone.0244744 (PMC7774861; doi:10.1371/journal.pone.0244744)
Supplement: S1 Table — (DOCX) [file pone.0244744.s001.docx]

S1 Table: Causes of death after liver transplant

|  | White | Black | Hispanic | Asian | Others | Total |
| --- | --- | --- | --- | --- | --- | --- |
| Cardiovascular | 1980 | 283 | 357 | 99 | 29 | 2748 |
|  | (12.89%) | (12.97%) | (14.75%) | (13.96%) | (13.06%) |  |
| Respiratory failure | 832 | 90 | 101 | 26 | 15 | 1064 |
|  | (5.42%) | (4.12%) | (4.17%) | (3.67%) | (6.76%) |  |
| Graft failure | 1550 | 356 | 272 | 68 | 25 | 2271 |
|  | (10.09%) | (16.32%) | (11.24%) | (9.59%) | (11.26%) |  |
| Hemorrhage | 388 | 79 | 73 | 27 | 9 | 576 |
|  | (2.53%) | (3.62%) | (3.02%) | (3.81%) | (4.05%) |  |
| Malignancy | 2688 | 217 | 379 | 159 | 33 | 3476 |
|  | (17.5%) | (9.95%) | (15.65%) | (22.43%) | (14.86%) |  |
| Immunosuppressive drug related | 12 | 1 | 2 | 0 | 0 | 15 |
|  | (0.08%) | (0.05%) | (0.08%) | (0%) | (0%) |  |
| Renal failure | 309 | 39 | 40 | 12 | 2 | 402 |
|  | (2.01%) | (1.79%) | (1.65%) | (1.69%) | (0.9%) |  |
| Operative | 1551 | 270 | 278 | 73 | 34 | 2206 |
|  | (10.1%) | (12.37%) | (11.48%) | (10.3%) | (15.32%) |  |
| Infection | 1888 | 313 | 326 | 79 | 24 | 2630 |
|  | (12.29%) | (14.34%) | (13.47%) | (11.14%) | (10.81%) |  |
| Others | 4161 | 534 | 593 | 166 | 51 | 5505 |
|  | (27.09%) | (24.47%) | (24.49%) | (23.41%) | (22.97%) |  |
| Total | 15359 | 2182 | 2421 | 709 | 222 | 20893 |
