# Supplementary material for: Liver transplant waitlist removal, transplantation rates and post-transplant survival in Hispanics
Source: PLoS One. 2020 Dec 31;15(12):e0244744. doi: 10.1371/journal.pone.0244744 (PMC7774861; doi:10.1371/journal.pone.0244744)
Supplement: S2 Table — (DOCX) [file pone.0244744.s002.docx]

S2 Table: Patients’ characteristics comparison between Hispanics and Non-Hispanics in matched cohort

| Variable | Non-Hispanic (N= 15267) | Hispanic (N= 15267) | P value |
| --- | --- | --- | --- |
| Region |  |  | 0.890 |
| 1 | 542 (3.55%) | 544 (3.56%) |  |
| 2 | 946 (6.20%) | 907 (5.94%) |  |
| 3 | 1985 (13.00%) | 1927 (12.62%) |  |
| 4 | 2745 (17.98%) | 2757 (18.06%) |  |
| 5 | 4961 (32.49%) | 5022 (32.89%) |  |
| 6 | 265 (1.74%) | 248 (1.62%) |  |
| 7 | 937 (6.14%) | 932 (6.10%) |  |
| 8 | 753 (4.93%) | 780 (5.11%) |  |
| 9 | 1569 (10.28%) | 1620 (10.61%) |  |
| 10 | 298 (1.95%) | 282 (1.85%) |  |
| 11 | 266 (1.74%) | 248 (1.62%) |  |
| Age, mean ± SD | 54.02 ± 10.83 | 54.00 ± 10.36 | 0.855 |
| Female | 5626 (36.85%) | 5558 (36.41%) | 0.419 |
| Body Mass Index, mean ± SD | 29.29 ± 6.20 | 29.34 ± 5.75 | 0.416 |
| Meld Score, mean ± SD | 18.00 ± 9.70 | 17.97 ± 9.26 | 0.758 |
| Highest education |  |  | 0.642 |
| Grade school (0-8) | 1946 (12.75%) | 1975 (12.94%) |  |
| High school (9-12) or GED | 8243 (53.99%) | 8175 (53.55%) |  |
| College/Technical school | 3100 (20.31%) | 3186 (20.87%) |  |
| Associate / Bachelor degree | 1505 (9.86%) | 1483 (9.71%) |  |
| Post-college degree | 473 (3.10%) | 448 (2.93%) |  |
| Insurance |  |  | 0.130 |
| Private | 7032 (46.06%) | 7054 (46.20%) |  |
| Public-Medicaid | 3941 (25.81%) | 3991 (26.14%) |  |
| Public-Medicare | 3504 (22.95%) | 3531 (23.13%) |  |
| Public-Others | 590 (3.86%) | 519 (3.40%) |  |
| Others | 200 (1.31%) | 172 (1.13%) |  |
| Work for income | 2927 (19.17%) | 2883 (18.88%) | 0.521 |
