# Supplementary material for: Liver transplant waitlist removal, transplantation rates and post-transplant survival in Hispanics
Source: PLoS One. 2020 Dec 31;15(12):e0244744. doi: 10.1371/journal.pone.0244744 (PMC7774861; doi:10.1371/journal.pone.0244744)
Supplement: Attachment — Submitted filename: Response to reviewers PONE-D-20-33490.docx [file pone.0244744.s003.docx]

PONE-D-20-33490: Response to reviewers:

Reviewer #1: This retrospective cohort analysis confirmed that there are significant racial disparities in waitlist removal rates due to death/deterioration, transplantation rates and post liver transplant
mortality, especially in Hispanic. This paper is very well written and it seems that there is no problem in general.

I have some comments.

1. The waitlist removal group is consisted only due to death or deterioration + due to liver transplantation? Or is there any cause of waitlist removal cause?
**Response: Few patients were removed from waitlist because of improved condition and other reasons as summarized below. We tried to separate removal due to improved conditions and other reasons as another competing event, but the results were similar because of smaller numbers.**

|  | **race(Race)** | | | | | |
| --- | --- | --- | --- | --- | --- | --- |
|  | **White** | **Black** | **Hispanic** | **Asian** | **Others/Unknown** | **Total** |
| **Still waiting (%)** | 6910 (6.3) | 717 (5.5) | 1954 (8.4) | 545 (7.8) | 155 (8.0) | 10281 |
| **Died/Deteriorated/too sick (%)** | 29393 (26.8) | 3360 (25.8) | 7243 (31.2) | 1696 (24.3) | 555 (28.6) | 42247 |
| **Transplanted (%)** | 63581 (58.0) | 7847 (60.3) | 11758 (50.6) | 3912 (56.1) | 1035 (53.3) | 88133 |
| **Improved/other reasons (%)** | 9769 (8.9) | 1096 (8.4) | 2268 (9.8) | 827 (11.9) | 197 (10.1) | 14157 |
| **Total** | 10965 | 1302 | 23223 | 6980 | 1942 | 154818 |

2. In the discussion p.12, the authors mentioned that when they adjusted weight list removal for socioeconomic differences using surrogate markers such as education, insurance or employment status, the differences in waitlist removal due to death/deterioration disappeared, but disparity in transplantation remained more or less unchanged. Why the disparity in transplantation remained more or less unchanged? What causes disparity in transplantation?

**Response: There have been many discussions and review articles on this topic. We have cited one of our own review on this topic (#1)**

1. **Nguyen GC, Thuluvath PJ. Racial disparity in liver disease: Biological, cultural, or socioeconomic factors. Hepatology 2008;47(3): 1058-1066.**

Reviewer #2: Thuluvath, et al. investigated risk analysis for waitlist removal of death/deterioration and transplantation by multivariate analysis, using UNOS data of more than 150,000. They found that Hispanics had higher waitlist removal due to death/deterioration and lower transplant rates than non-Hispanics. Also, Hispanics did have better graft and patient survival than non-Hispanics. When covariables including socioeconomic status was adjusted, the higher risk in removal of death/deterioration disappeared, but not for transplantation. They discussed that these results were observational and could not find good reasons. This is interesting manuscript, but lacks data and explanations to support their theory.

Major
1. The numbers of patients are not compatible. For example, the number patients who received transplant supposed to be 154818 x 0.569 =88,091. Looking at table 3, the number of the patients were 57864+7221+10725+3573+985=80,368. Ten percent of the patient`s data are missing. These incompatibilities can lose quality of statistics of this study.

**Response: This was a complete oversight (wrong table included) on our side, the correct table is included in the revised manuscript. The total number is 88,133.**

| Variable | White | Black | Hispanic | Asian | Others |
| --- | --- | --- | --- | --- | --- |
|  | (N=63581) | (N=7847) | (N=11758) | (N=3912) | (N=1035) |
| **Recipients’' characteristics at transplantation:** | | | | | |
| Age, Mean (SD) | 54.8 (10.20) | 51.6 (12.18) | 53.8 (10.53) | 55.2 (10.94) | 52.2 (11.01) |
| Recipient gender: Female | 20226 (32%) | 3282 (42%) | 4186 (36%) | 1269 (32%) | 423 (41%) |
| Recipient BMI, Mean (SD) | 28.6 (5.70) | 28.5 (6.18) | 28.9 (5.60) | 25.0 (4.43) | 29.7 (5.96) |
| Morbidly Obese | 2466 (4%) | 378 (5%) | 518 (4%) | 21 (1%) | 68 (7%) |
| ALBUMIN, Mean (SD) | 3.1 (0.71) | 2.9 (0.77) | 3.0 (0.75) | 3.3 (0.81) | 3.0 (0.72) |
| TOTAL BILIRUBIN (MG/DL) , Mean (SD) | 8.0 (10.45) | 10.0 (11.61) | 10.2 (12.47) | 8.7 (12.49) | 10.1 (12.28) |
| Serum Creatinine, Mean (SD) | 1.4 (1.03) | 1.5 (1.20) | 1.4 (1.10) | 1.2 (0.95) | 1.4 (0.97) |
| INR, Mean (SD) | 1.9 (1.31) | 2.1 (2.07) | 2.0 (1.28) | 1.8 (1.50) | 2.1 (1.20) |
| Meld score, Mean (SD) | 21.3 (10.23) | 22.9 (11.18) | 23.2 (11.40) | 18.9 (12.18) | 23.4 (11.23) |
| Ascites is moderate at transplant | 16883 (27%) | 1754 (22%) | 3256 (28%) | 633 (16%) | 291 (28%) |
| Encephalopathy is 3-4 at transplant | 6593 (10%) | 921 (12%) | 1304 (11%) | 364 (9%) | 156 (15%) |
| KPS categories at transplant |  |  |  |  |  |
| 10 - 40 | 17497 (28%) | 2442 (31%) | 4038 (34%) | 953 (24%) | 354 (34%) |
| 50 - 70 | 22145 (35%) | 2543 (32%) | 3959 (34%) | 1156 (30%) | 361 (35%) |
| 80 - 100 | 14712 (23%) | 1767 (23%) | 2201 (19%) | 1250 (32%) | 228 (22%) |
| Missing | 9227 (15%) | 1095 (14%) | 1560 (13%) | 553 (14%) | 92 (9%) |
| Child-Pugh categories at transplant |  |  |  |  |  |
| A:5 or 6 points | 6664 (12%) | 1003 (14%) | 1165 (11%) | 1177 (33%) | 115 (12%) |
| B:7-9 points | 17372 (30%) | 1765 (24%) | 2846 (27%) | 908 (25%) | 215 (22%) |
| C:>9 points | 33780 (58%) | 4449 (62%) | 6705 (63%) | 1481 (42%) | 655 (66%) |
| Medical condition |  |  |  |  |  |
| In intensive care unit | 6495 (11%) | 1107 (15%) | 1816 (17%) | 557 (16%) | 154 (16%) |
| Hospitalized not in ICU | 9866 (17%) | 1327 (18%) | 2207 (21%) | 465 (13%) | 190 (19%) |
| Not hospitalized | 41448 (72%) | 4783 (66%) | 6685 (62%) | 2545 (71%) | 639 (65%) |
| **Donors' characteristics:** | | | | | |
| DONOR AGE (YRS), Mean (SD) | 41.9 (16.56) | 40.4 (16.13) | 41.3 (16.79) | 41.1 (17.69) | 40.7 (16.70) |
| Donor gender: Female | 23177 (40%) | 3035 (42%) | 4457 (42%) | 1623 (45%) | 424 (43%) |
| CREATININE , Mean (SD) | 1.6 (1.70) | 1.6 (1.74) | 1.6 (1.73) | 1.6 (1.73) | 1.6 (1.81) |
| BILIRUBIN , Mean (SD) | 0.9 (1.04) | 0.9 (1.39) | 1.0 (1.48) | 0.9 (1.16) | 0.9 (1.45) |
| Donor DIABETES | 6311 (11%) | 808 (11%) | 1182 (11%) | 372 (11%) | 97 (10%) |
| Calculated Donor BMI, Mean (SD) | 27.6 (6.31) | 27.3 (6.16) | 27.0 (5.94) | 26.0 (5.62) | 27.4 (6.43) |
| DRI, Mean (SD) | 1.8 (0.45) | 1.8 (0.42) | 1.8 (0.45) | 1.9 (0.46) | 1.8 (0.45) |

2. The authors should have better explanation regarding better graft and patient survival in Hispanics, not just to conclude with “biological reasons”. What are the biological reasons? Since Hispanics had higher MELD score with higher dialysis rates, lower KPS, etc, this population should have worse graft and patient survival. We can’t agree with the explanation for “it is plausible that ……“, since Hispanics had higher risks than other races.

**Response: we have adjusted for these variables in the multivariate analysis.**
3. Showing Figure 3 A and 3B without matching SEC does not make sense. It is important to show the graft and patient survival curves after matching with covariables including SEC. We think “Sensitivity analysis” paragraph and “Impact of socioeconomic status….” paragraphs should be combined to one. Also, it is meaningless to show duplicates in Table 2 and Table 5. They should only show Table 5, with reorganized paragraphs in the Result section.

**Response:** **We redid matching based on region, age, sex, BMI, Meld score, highest education, insurance and employment, and regenerated figure 3A and 3B, the conclusion is similar.**

**We removed table 2 and only kept table 5 (table 2 in the revised MS)**

**Paragraphs reorganized as suggested.**

4. The authors need to discuss why malignancy is the major cause of death for Hispanics.

**Response: it is highest in Asians followed by whites (see supplementary table 1).**

Minor
1. We could not find the data that explains “more Asians received poor-quality graft (defined as DRI>2) than whites ……..” in Table 1.

**Response: although the median DRI was similar, a higher proportion of Asians received DRI >2. We have shown that data in the result section (page 10, last sentence of 1^st^ paragraph).**

2. Introduction and Result section, multivariate analyses for not “patient survival” and “graft survival”, we think “patient mortality” and “graft failure”.

**Response: we revised the table as suggested. Thanks.**
3. What does 93.2% mean at introduction section (line 8)? The author should have more care for adding this number, or exclude this from the manuscript.

**Response: that was accidentally inserted and we should have done better proof reading. Thank you.**

4. Page 9, in multivariate analyses for graft and patient, “gender” should be included as a risk factor.

**Response: gender added**
5. Page 12, line 18, “weight list”=>”waitlist”

**Response: changed as suggested**
